# Ultrafast micro/nano-manufacturing of metastable materials for energy

**DOI:** 10.1093/nsr/nwae033

**Published:** 2024-01-27

**Authors:** Xiaoya Cui, Yanchang Liu, Yanan Chen

**Affiliations:** School of Materials Science and Engineering, Key Laboratory of Advanced Ceramics and Machining Technology (Ministry of Education), and Tianjin Key Laboratory of Composite and Functional Materials, Tianjin University, Tianjin 300072, China; Ministry of Education Key Laboratory of Protein Sciences, School of Life Sciences, Tsinghua University, Beijing 100084, China; School of Materials Science and Engineering, Key Laboratory of Advanced Ceramics and Machining Technology (Ministry of Education), and Tianjin Key Laboratory of Composite and Functional Materials, Tianjin University, Tianjin 300072, China; School of Materials Science and Engineering, Key Laboratory of Advanced Ceramics and Machining Technology (Ministry of Education), and Tianjin Key Laboratory of Composite and Functional Materials, Tianjin University, Tianjin 300072, China

**Keywords:** high-temperature shock technique, defect engineering, metastable phase, kinetic modulation, non-equilibrium micro/nano-manufacturing

## Abstract

The structural engineering of metastable nanomaterials with abundant defects has attracted much attention in energy-related fields. The high-temperature shock (HTS) technique, as a rapidly developing and advanced synthesis strategy, offers significant potential for the rational design and fabrication of high-quality nanocatalysts in an ultrafast, scalable, controllable and eco-friendly way. In this review, we provide an overview of various metastable micro- and nanomaterials synthesized via HTS, including single metallic and bimetallic nanostructures, high entropy alloys, metal compounds (e.g. metal oxides) and carbon nanomaterials. Note that HTS provides a new research dimension for nanostructures, i.e. kinetic modulation. Furthermore, we summarize the application of HTS—as supporting films for transmission electron microscopy grids—in the structural engineering of 2D materials, which is vital for the direct imaging of metastable materials. Finally, we discuss the potential future applications of high-throughput and liquid-phase HTS strategies for non-equilibrium micro/nano-manufacturing beyond energy-related fields. It is believed that this emerging research field will bring new opportunities to the development of nanoscience and nanotechnology in both fundamental and practical aspects.

## Teaser

High-temperature shock (HTS) technique, as a non-equilibrium synthesis strategy, offers significant potential for the rational design and fabrication of high-quality metastable materials in an ultrafast, scalable, controllable, and eco-friendly way.

## INTRODUCTION

Increasing concerns regarding global warming, environmental pollution and the energy crisis have prompted extensive exploration of sustainable energy strategies. Nevertheless, economic growth decouples both energy consumption and CO_2_ emissions [1,2]. Full decarbonization and a carbon-neutral society with an effective and environmentally friendly energy system remains a great challenge. For instance, fuel cells, as a promising energy-storage system, have unique properties such as safety, eco-friendliness and superior activity [3,4]. However, the stability of fuel cells still needs to be improved, especially at high currents. This improvement would be of great significance for industrial applications. Importantly, the manufacturing process for micro- and nanocatalysts has encountered great obstacles, such as high surface tension, particle agglomeration, poor activity and stability, complicated fabrication, low producing efficiency, high cost and limited intrinsic structures [5]. Therefore, great effort should be devoted to developing effective, cost-efficient and compelling strategies to synthesize stable and high-performing novel functional materials with unique structures and properties, such as metastable materials and amorphous or heterophase nanostructures, for advancing renewable energy storage and alleviating global warming.

Metastable nanomaterials with abundant defects, including high entropy alloys (HEAs), show great potential in catalytic procedures with high performance and stability, since the electronic structure is strongly associated with the crystal configuration. Taking HEAs as an example, the main physical parameters contributing to the metastability include: (i) Complex composition. HEAs have a complex and high number of alloying elements, leading to a diverse atomic arrangement. This complexity often results in a lack of equilibrium phases, contributing to metastability. (ii) Entropy stabilization. The high entropy in HEAs plays a crucial role in stabilizing the alloy in a metastable state. The entropy-driven stabilization can hinder the transformation to more thermodynamically stable phases. (iii) Configurational entropy. The configurational entropy, associated with the disorder in the arrangement of atoms, also contributes to the metastable nature of HEAs. Normally, metastable materials are produced under extreme conditions involving high temperature, high pressure, electron irradiation and other harsh kinetic conditions [4,6–11]. Currently, the manufacturing of metastable nanomaterials encounters formidable challenges, such as limited intrinsic structures, particle agglomeration, poor activity and stability, complicated fabrication processes, and low producing efficiency, owing to the thermodynamic instability of metastable materials and the heuristic principles for preparing metastable materials. Moreover, conventional methods, such as rapid quenching, mechanical alloying, pulse laser deposition, hydrothermal synthesis and the sol-gel method, are employed for producing various metastable materials as well [12]. However, these methods encounter challenges such as limited particle size, high energy consumption, complex equipment requirements, long processing time, difficulties in achieving uniform size and distribution, and environmental concerns. Recently developed ultrafast synthesis methods, such as the microwave (MW) method, spray pyrolysis (SP) and laser ablation in liquid (LAL), garnered much attention from academic researchers [13–15]. In particular, the MW method boasts high energy efficiency, with rapid heating (160 K s^−1^) and cooling (450 K s^−1^) rates [13]. However, its limited penetration depth in highly dielectric materials like carbon materials restricts it to small-scale production. The SP method offers a short preparation period and good production continuity, but drawbacks include the absence of a rapid quenching process and slow reaction kinetics, potentially leading to the phase separation of nanomaterials [14]. The key technique of the LAL method involves using a high-energy laser to heat the target materials in the liquid phase, featuring a super-quenching effect with a cooling rate of up to 10^10^ K s^−1^ [15]. LAL demonstrates advantages such as being environmentally friendly without byproduct generation, universality across various materials and solvents, and the capability to control the phase, size and morphology of nanomaterials. However, LAL might be confined to small-scale applications with potential contamination risks. Note that the atomic rearrangement occurring under high-temperature heating can modulate the crystal structures of nanomaterials, which is beneficial for the formation of metastable nanomaterials [16].

The ultrafast high-temperature shock (HTS) method, proposed by Chen *et al*. in 2016 [17], provides a simple, flexible and high-throughput manufacturing platform for synthesizing metastable materials, including single metals, bimetals, HEAs and metal compounds like oxides, carbides, nitrides and sulfides [6,18]. By using the homemade set-up to conduct this non-equilibrium Joule heating process, Al nanoparticles on a reduced graphene oxide (rGO) substrate were synthesized for application in the field of batteries and catalysts. Importantly, the defects in rGO effectively prevented the particles from agglomeration and surface oxidation, yielding well-dispersed nanoparticles with refined sizes. Specifically, by using instant ultrahigh energy input, the HTS system provided an ultrafast Joule heating (up to 3000 K) on reaction precursors (e.g. metal salts, bulk metals and carbon compounds) which were decomposed into metal atoms, followed by nucleation and a grain growth process, resulting in the formation of metastable materials. Impressively, the heating and cooling rates during HTS can reach 10^5^ K/s and 10^4^ K/s, respectively, owing to its high-power density. This introduces a novel dimension for the design of nanostructures, i.e. kinetic modulation, which proves beneficial for the synthesis of defect-rich, new crystal phase nanomaterials and HEAs. In addition, the dwelling time in this process can be as short as 50 ms, effectively preventing particle coarsening and crystal phase transformations under high temperatures. Due to the ultrafast cooling rate, the high entropy (Δ*S*) structure can be reserved. Therefore, the aforementioned ultrafast synthesis strategy can overcome the limitations of traditional methods, enabling the rational design of metastable catalysts with tunable compositions, structures, crystal phases and related physiochemical properties [19,20]. These distinctive features of the HTS method create non-equilibrium reaction conditions, rendering it feasible for the synthesis of homogenous metastable materials. Compared with conventional methods, HTS demonstrates great potential for industrial applications. The kinetic mechanism involves considering the thermodynamics and kinetics of reactions during HTS synthesis. While the specifics may vary depending on the exact method used and the composition of the HEA, the general principles involve exploiting the non-equilibrium conditions to produce metastable phases with unique properties. Currently, the HTS technique primarily focuses on solid-state reduction from precursors to products [21]. However, there is still room for improvement to enhance its versatility for different reaction systems and high-throughput screening and synthesis of catalysts. Importantly, incorporating factors such as capping agents, reducing agents, solution composition and pressures in the HTS process would contribute to a broader and more universally applicable approach, promoting the development of effective metastable catalysts.

**Figure 1.**
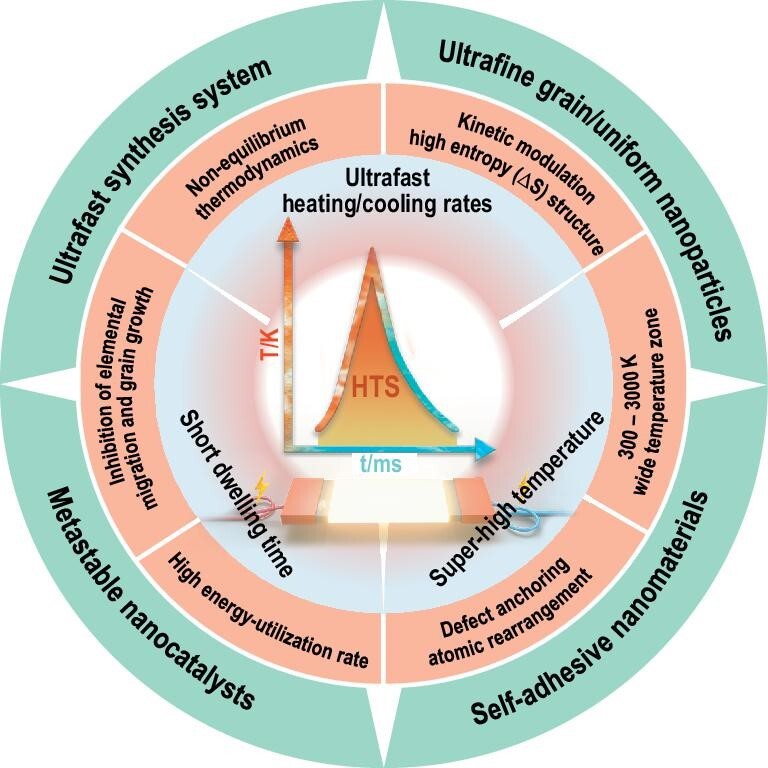
Schematic overview of the HTS technique illustrated in this review. Characteristics and applications of this method in the manufacturing of metastable nanomaterials are highlighted.

Herein, we systematically introduce recent advances in the manufacturing of metastable materials via high-throughput and environmentally friendly HTS methodology in various electrocatalytic, energy-storage and conversion applications. The significant innovations and potential of HTS, as well as the kinetic mechanisms of metastable materials, are discussed. Importantly, perspectives on the HTS strategy for high-throughput manufacturing of metastable materials, liquid phase HTS synthesis and advanced material characterizations are outlined (Fig. 1). We believe this review will provide valuable insight into the structural engineering and kinetic modulation of metastable materials with outstanding performance.

## HTS TECHNIQUE FOR ULTRAFAST MANUFACTURING

The HTS technique, initially proposed by Chen *et al.* in 2016, has emerged as an advanced and efficient method for the ultrafast manufacturing of micro- and nanomaterials [17]. This methodology has yielded a series of excellent catalysts, including metal and semiconductor nanomaterials, alloys, metal compounds, high entropy alloys and compounds, ceramics, and carbon materials. Meanwhile, the HTS technique has been extended by researchers to synthesize new nanoalloys featuring a uniform distribution of multiple immiscible elements, indicating its potential for high-throughput synthesis and industrial-scale production of nanomaterials.

Inspired by HTS, Luong *et al*. applied the flash Joule heating method to convert economically accessible carbon sources into graphene, achieving a high yield of 80%–90% within a short time [22]. Yao *et al*. introduced a periodic electric pulse step within the HTS technique, wherein the electric pulse is activated for 55 ms and deactivated for 550 ms successively [23]. Using the pulse HTS approach, the production of single-atomic catalysts can be facilitated. More recently, Wang *et al*. integrated the HTS technique with the SP method, employing a carbon wood microchannel reactor capable of reaching temperatures up to 2000 K to replace conventional heating furnaces [24]. In this configuration, atomized precursor droplets undergo rapid heating in the microchannel reactor within milliseconds, enabling the ultrafast continuous synthesis of nanomaterials without agglomeration, growth, or phase separation. Additionally, Yao *et al*. developed a high-throughput HTS method utilizing a scanning droplet cell for the electrochemical rapid screening of catalysts [25]. In summary, the HTS technique, pioneered by Chen *et al*., represents an intricate and powerful approach for the ultrafast fabrication of micro- and nanomaterials, and has been applied in many energy-related fields. Its expansion into diverse methodologies, such as flash Joule heating, periodic electric pulse steps and integration with spray pyrolysis, underscore its versatility and potential for transformative advancements in high-throughput synthesis, leading to a paradigm shift towards efficient and scalable nanomaterial production in the pursuit of cutting-edge scientific and technological innovation.

HTS has several typical characteristics: ultrafast heating or cooling rates, short dwelling time and superhigh temperatures. Notably, the HTS process demonstrates high local temperatures across a wide range, from 300 K to 3000 K [6]. In the HTS process of nanomaterials, the reaction takes place in a superhigh-temperature zone, where the overheating of this reaction system compels metal precursors and bulk materials to undergo rapid decomposition, leading to extensive nucleation and defect anchoring. Nevertheless, the short dwelling time at high temperatures effectively inhibits grain growth and elemental migration. Simultaneously, the ripening and aggregation of nanoclusters or nanoparticles are also prevented. Subsequently, the high cooling rate induces significant undercooling, providing sufficient kinetic conditions to dominate the thermodynamic mixed state, and thereby results in the formation of fine-grained and metastable nanostructures. Note that defects such as vacancies, dislocations, strains, twin boundaries and stacking faults are effectively maintained within these nanostructures. Consequently, the electronic structure of active sites can be regulated, enhancing the interaction between adsorption intermediates and active sites, and thereby promoting the electrocatalytic performance. This effective kinetic modulation of nanostructures has great significance in terms of developing the structure-based design of novel materials with controlled phases, ultrafine sizes, and rich defects and strains [26–28].

**Figure 2.**
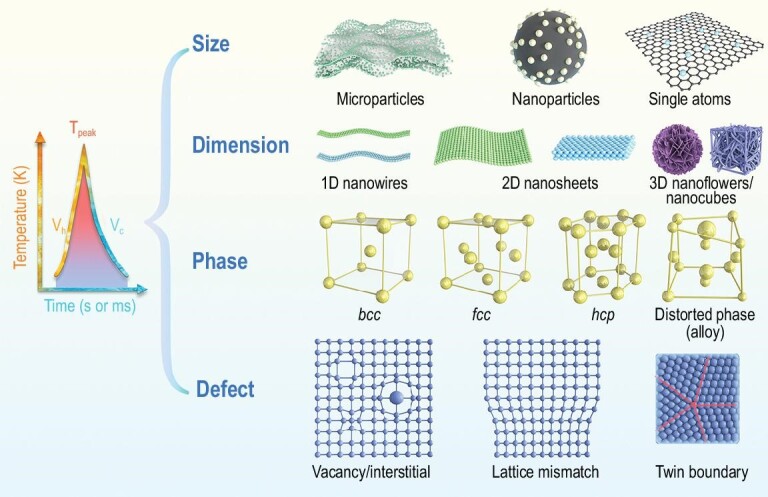
Schematic illustration of HTS for the structural engineering of nanoparticles. Rational design of nanoparticles from the aspects of size (micro-/nanoparticle and single atom), dimension (0D, 1D, 2D and 3D), phase (conventional *bcc, fcc* and *hcp* phases, novel phase, and distorted phase) and defect (vacancy/interstitial defects, lattice mismatch and boundary).

Specifically, solid-state synthesis applies metal precursors such as solid-state micromaterials (e.g. metals, semiconductors and composites) and metal salt solutions. To effectively construct ultrafine nanomaterials, conductive supporting substrates such as carbon nanofibers (CNFs), rGO and carbonized biomass (e.g. wood) are applied. These substrates facilitate the preparation of evenly dispersed and uniform nanoparticles from the aforementioned metal precursors. To date, the solid-state HTS strategy has been applied to prepare various nanomaterials (Fig. 2), including single metals and semiconductors (e.g. Si, Al, Au, Ag, Pt, Pd, Ru, Ir, Ni and Sn) [17,29–32], multimetallic alloys (e.g. PtFe, PdNi, NiFe, CuNi, IrNi and CuAg) [33–37], HEAs (e.g. PtPdRhRuCe, CoMoFeNiCu, AuAgPtPdCu and PtPdCoNiFeCuAuSn) [26,38], single atoms (e.g. Ru, Pt, Pd and Co) [23,39] and metallic compounds (e.g. SiC, CoS, FeS_2_, Co_3_O_4_, MoS_2_, CoFeP_x_, LiMn_2_O_4_, LiCoO_2_, LiFePO_4_ and Li-rich layered oxide/NiO heterostructures) [40–45].

This non-equilibrium kinetic process under extreme conditions exhibits ultrahigh energy conversion efficiency, high local temperature and a wide temperature range, which is beneficial for the synthesis of well-dispersed, uniform, stable and ultrafine-sized crystalline or metastable nanocatalysts and assemblies in an instant synthesis process. HTS induces abundant defects and heterointerfaces within nanocatalysts, which can not only generate rich catalytic sites and metastable phases, but also enhance the chemical bond between nanocatalysts and substrates, improving their mechanical stability due to the ultrahigh reducing temperature [46].

## STRUCTURAL ENGINEERING OF METASTABLE NANOMATERIALS BY HTS

Controllable synthesis of inorganic nanostructures with low crystallinity, such as metastable phases, amorphous phases and amorphous-crystalline heterophases, is fundamentally important for engineering crystal phases and structures beyond ordered atomic arrangements [27,47,48]. Metastable materials exhibit features such as high entropy, disordered or short-range ordered atomic arrangements, and abundant structural defects. Recently, metastable nanostructures have emerged as a novel kind of functional material with fantastic applications in catalysis, lithium-ion batteries (LIBs), electronic devices, etc. Importantly, metastable nanomaterials are usually synthesized under extreme external heat or pressure, limiting high-throughput fabrication and industrial application. Due to the characteristics of the HTS technique, including ultrafast heating and cooling rates, short dwelling time and a superhigh reaction temperature zone, kinetic control over the structural engineering of metastable materials can be easily realized. Therefore, the HTS strategy shows great potential for the preparation of metastable nanomaterials, including metal nanostructures, compounds and carbon nanomaterials, with tunable crystal structures, defects, lattice strains and morphologies. However, for the rational design of metastable materials, crucial considerations involve precise control over parameters during the HTS process, encompassing temperature, pressure and duration, to govern atomic arrangement and phase engineering. Importantly, controlling the formation of metastable materials by the HTS method requires careful consideration of reaction kinetics, including nucleation and growth processes. The integration of *in-situ* diagnostics, advanced characterization techniques and computational modeling is essential to unravel the dynamic processes and provide insight into the resulting material properties. Moreover, the application of HTS in energy-related fields demands a focus on specific application requirements, such as catalysis and energy-storage materials. The scalability, reproducibility and environmental impact of the HTS method are crucial in the design and implementation of large-scale production of energy-related materials. In summary, the successful design of HTS for metastable materials in energy applications involves a nuanced integration of thermodynamics, kinetics and advanced characterization techniques, ensuring a robust and versatile synthesis of high-performance catalysts.

### Metal nanostructures

#### Monometallic nanostructures

Lu *et al*. fabricated stable Co@N-C core-shell structures by encapsulating Co nanoparticles in the N-doped hollow porous carbon nanofibers (PCNFs) using HTS [49]. The as-prepared Co@N-C/PCNF, featuring refined crystal size, modulated local electrical structures and a variety of chemical structures, showed excellent electrocatalytic performance for the oxygen evolution reaction (OER) and oxygen reduction reaction (ORR) [49]. The overpotential at 10 mA cm^−2^ was only 289 mV for OER, while the half-wave potential was 0.85 V for ORR. In addition, they fabricated Co N-C/PCNF-based flexible Zn-air batteries (ZABs) with a superior specific capacity of 292 mW cm^−2^, great cycle life performance and high flexibility, potentially advancing the development of wearable devices.

**Figure 3.**
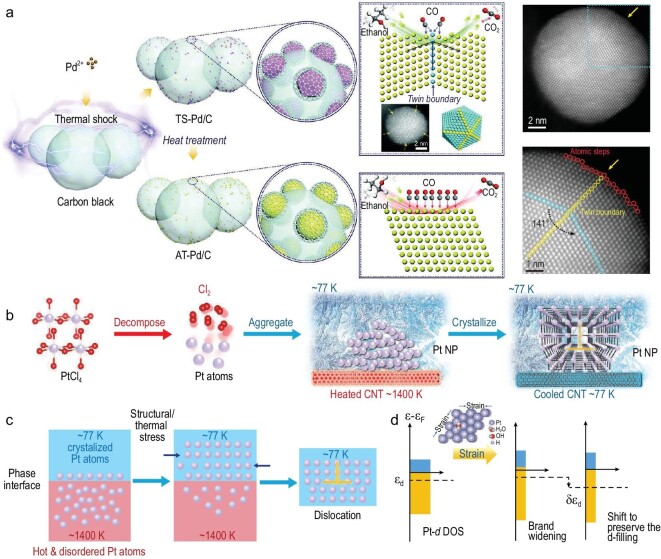
Manufacturing of metastable monometallic nanostructures. (a) Schematic explanation of the manufacturing process of defect-rich TS-Pd/C with TBs and ASs. The annealed AT-Pd/C with fewer defects is used as the reference. The electrocatalytic EOR mechanism is illustrated in the inset. Reproduced with permission from [50]. Copyright 2022, Wiley-VCH GmbH. (b) Schematic illustration of the synthesis of Pt nanoparticles with rich dislocations by HTS equipped with a liquid nitrogen cooling system. (c) The dislocation formation process during the reduction of Pt, resulting from the structural and thermal stress. The fast-cooling rate arising from liquid nitrogen kinetically generates and fixes the dislocations inside Dr-Pt on the carbon nanotube (CNT). (d) Pt *d*-band variation due to the compressive strain. Reproduced with permission from [32]. Copyright 2021, Wiley-VCH GmbH.

Moreover, metastable Pd nanoparticles on carbon substrates with high-density defects, including twin boundaries (TBs) and atomic steps (ASs), were synthesized via the solid-state HTS technique within 3 s (Fig. 3a) [50]. The as-prepared Pd nanoparticles, uniformly distributed on carbon black, i.e. thermal shock Pd/C (TS-Pd/C), showed an average diameter of 6.8 nm. In this HTS process, the peak temperature could reach 1601 K, with superior heating and cooling rates of ∼1.0 × 10^3^ and 2.0 × 10^2^ K s ^−1^, respectively. By heat treatment in argon, the TS-Pd/C was transformed into annealing-treated Pd/C (AT-Pd/C), involving structure relaxation and atomic rearrangement [51]. Molecular dynamics (MD) simulation confirmed the formation of TBs during the rapid cooling process. Moreover, in this process, the generation of TBs was also prompted by the local stresses generated due to the temperature gradient extending from the center to the surface of the nanoparticle. Impressively, the TS-Pd/C showed advanced activity, stability and CO tolerance for the ethanol oxidation reaction (EOR). First-principles calculations explained that the high-density defects, including TBs and ASs, spatially separated the optimized adsorption sites for CO and OH, effectively reducing the ratio of poisoned active sites and enhancing the activity for EOR.

To efficiently construct rich dislocations in single metals, kinetic modulation is essential to overcome the challenges posed by the small size and high crystallinity inherent to single metals. Notably, liquid nitrogen can serve as a cooling medium during the HTS process, establishing a more extreme kinetic reaction system. This system can promote and retain dislocations, as well as generate strains within the single metal nanocatalysts. Recently, Liu *et al*. used liquid nitrogen as the cooling medium (≈77 K) during the HTS synthesis of Pt nanoparticles with rich dislocations (i.e. Dr-Pt) at a temperature of 1500 K for 20 ms (Fig. 3b) [32]. The dislocations were instantly induced during the crystallization process by structural and thermal stress, and then kinetically frozen inside the Dr-Pt nanoparticles due to the ultrafast cooling rate. Notably, the dislocations can induce strain, which downshifts the *d*-band center of Pt and weakens the adsorption energy between Pt and reaction intermediates during electrocatalysis, as shown in Fig. 3c and d. Dr-Pt showed a low overpotential of 25 mV at 10 mA cm^−2^ in a 1 M potassium hydroxide (KOH) solution and demonstrated excellent long-term stability for the hydrogen evolution reaction (HER).

#### Bimetallic alloys

Distinct from monometallic nanostructures, bimetallic nanomaterials show novel catalytic properties which cannot be obtained in their single counterparts [52,53]. Until now, it has been difficult to identify the origin of the superior performance of bimetallic systems, considering the limited access to homogeneous bimetallic alloys due to the inherent thermodynamic immiscibility of various elements. The non-equilibrium HTS strategy has great potential to address the thermodynamic immiscibility problem in bimetallic systems. Recently, bimetallic alloys have been manufactured via the HTS method, including binary immiscible alloys (e.g. Au-Ni, Cu-Ag and Rh-Au) and hybrid heterostructures (e.g. Ag/Co/C) with abundant surface-active sites, dislocations and grain boundaries [26,35,54,55].

**Figure 4.**
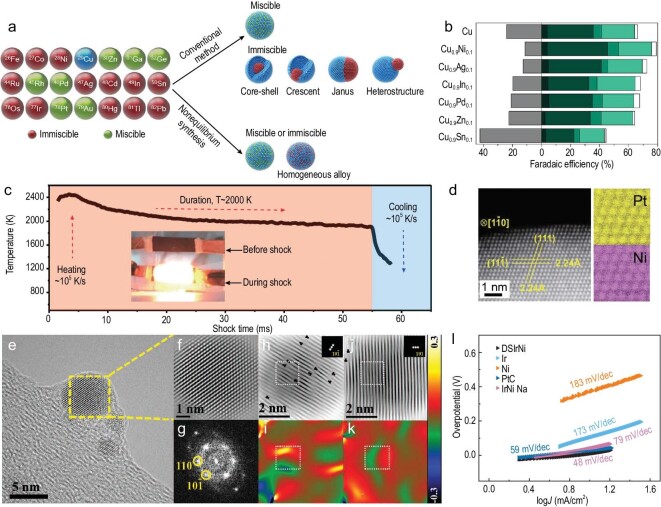
Synthesis of defect-rich bimetallic materials by the HTS method. (a) Bimetallic nanoparticle synthesis pathways via conventional and non-equilibrium strategies. Note that non-equilibrium synthesis, such as the HTS technique, can overcome the energy barriers to form homogeneously mixed bimetallic alloys (Cu and other metals), regardless of the thermodynamic miscibility. (b) Faradaic efficiency of Cu and distinct Cu_0.9_X_0.1_ bimetallic nanocatalysts at −0.70 ± 0.01 V vs. reversible hydrogen electrode (RHE). Reproduced with permission from [35]. Copyright 2020, AAAS. (c) Temporal evolution of temperature during the HTS process for 55 ms. (d) High-angle annular dark-field scanning transmission electron microscopy (HAADF-STEM) image of PtNi nanoalloy and the corresponding atomic elemental mappings. Reproduced with permission from [26]. Copyright 2018, AAAS. (e) High-resolution TEM (HRTEM) image of the IrNi nanoparticle. (f) The inverse fast Fourier transform (IFFT) image and (g) corresponding FFT pattern of the nanoparticle marked in the dash square region in (e). (h and j) The corresponding IFFT patterns showing abundant dislocations along (10$\bar{1}$) and (110), respectively. (i and k) Strain distribution of e*_xx_* and e*_xy_*, corresponding to the (10$\bar{1}$) and (110) planes, respectively. Reproduced with permission from [37]. Copyright 2020, Wiley-VCH GmbH. (l) Corresponding Tafel slopes of the catalysts.

Using the non-equilibrium HTS method, Yang *et al*. created a Cu-based bimetallic library of homogeneous alloyed nanoparticles, i.e. Cu_0.9_X_0.1_ (X = Ag, Ni, Sn, In, etc., which is immiscible with Cu at room temperature). This library showed excellent performance in carbon monoxide reduction [35]. Specifically, Cu_0.9_Ni_0.1_ showed a Faradaic efficiency of 76% for multicarbon chemicals at a current density of 93 mA cm^−2^ (Fig. 4a and b). Additionally, atomic-scale uniform PtNi nanoalloys were synthesized with the *fcc* phase by the carbothermal shock method at ∼2000 K (Fig. 4c and d) [26]. Furthermore, Liu *et al*. demonstrated that the HTS technique can *in-situ* induce the formation of dislocations within metastable IrNi alloy nanoparticles loaded on a carbon nanotube sponge, referred to as DSIrNi@CNTS (Fig. 4e–g). This phenomenon occurs as a consequence of the extremely fast heating and cooling process driven by the disparity in atomic radii [37]. Therefore, the strain effects were induced by numerous dislocations (Fig. 4h–k), which have been demonstrated to optimize the electronic structure of active sites by downshifting of the *d*-band center of Ir towards the Fermi level. Together with the synergistic effect of Ir and Ni elements and the carbon substrate, the binding energy of hydrogen was weakened, and thus, the metastable IrNi showed enhanced HER performance with an overpotential of 17 mV at 10 mA cm^−2^ and a Tafel slope of 48 mV dec^−1^ (Fig. 4l).

#### High entropy alloys

The HEA nanocatalyst, a simple solid solution with an atomic ratio of five or more kinds of elements ranging from 5% to 35%, can be regarded as a promising candidate for electrocatalytic reactions such as water splitting, fuel cells and CO_2_ conversion [56]. Due to its distinct and flexible composition, abundant active sites and high-entropy instinct structure, HEA shows superior activity, selectivity and stability. The interactions among the multiple components of HEA can be characterized into four groups of effects—thermodynamic high entropy, structural lattice distortion, kinetic sluggish diffusion and ‘cocktail’ effects—contributing to the unique advantages of HEAs in electrocatalytic reactions [57–59]. Specifically, due to the high entropy effect, HEAs can be synthesized with tunable compositions and regulated electronic and geometric structures, significantly enhancing their catalytic performance [60,61]. The lattice distortion and sluggish diffusion effects lead to the modification of atomic arrangements, inducing rich active sites with minimized local free energy on the surface of nanostructures. This attracts more reactants or intermediates, boosting electrocatalytic activity. It is difficult to prepare multimetallic alloys exceeding three elements using wet-chemical methods, due to the high energy required for the simultaneous reduction and mixing of multiple elements. Despite the fact that other conventional alloying methods such as printing- and lithography-based approaches may fabricate quaternary and even quinary nanostructures, these as-obtained nanostructures often undergo elemental migration and phase separation, especially for thermodynamically immiscible compositions [62,63]. To date, the investigation of advanced HEA catalysts with refined surfaces, morphologies, structures and crystal phases for electrocatalytic applications remains a significant challenge. The controllable design of multiple immiscible elements into ultrafine-sized and single nanoparticles with nanoscale size is a daunting task, and highly desirable for revealing the fundamental aspects of the structure–property relationship in electrocatalytic reactions, including water splitting (e.g. HER, HOR, OER and ORR) and CO_2_ conversion [26,64,65].

**Figure 5.**
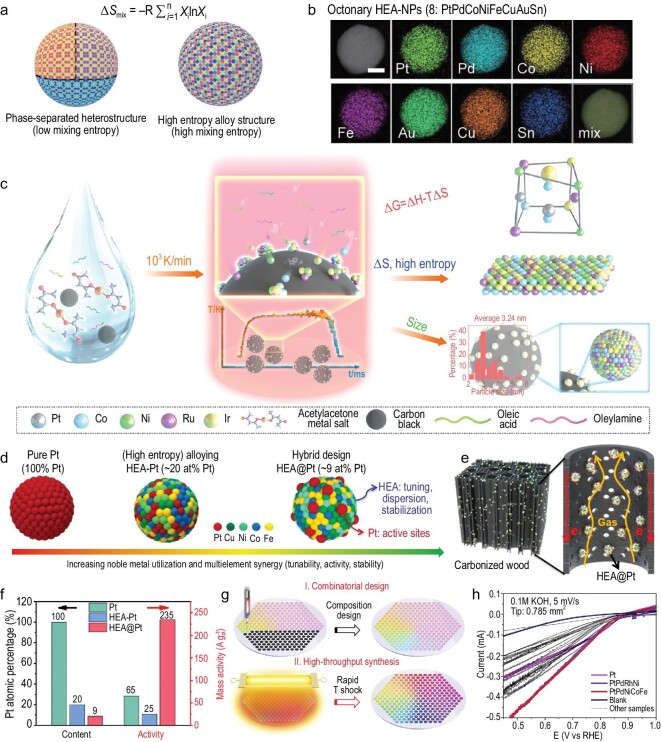
Structural engineering of HEAs using rapid Joule heating. (a) Structure schemes of phase-separated heterostructures (mild reduction procedure with slow kinetics) and solid-solution HEAs (carbothermal shock with fast kinetics). (b) Elemental mapping of an HEA nanoparticle with eight elements. Scale bar: 10 nm. Reproduced with permission from [26]. Copyright 2018, AAAS. (c) Schematic illustration of the synthetic process of PtCoNiRuIr/C via the high-temperature liquid shock (HTLS) method. Reproduced with permission from [67]. Copyright 2024, American Chemical Society. (d) Structure schemes of Pt, HEA-Pt and HEA@Pt nanoparticles. (e) Schematic illustration of HEA@Pt loaded on carbonized wood. (f) Pt content and mass activity comparisons of the aforementioned three nanomaterials. Reproduced with permission from [68]. Copyright 2022, Wiley-VCH GmbH. (g) Schematic illustration of the combinatorial design and high-throughput synthesis of multimetallic nanoclusters, including HEAs. (h) Catalytic ORR performance of PtPd-based multimetallic nanoclusters at a scan rate of 5 mV/s in 0.1 M KOH. Reproduced with permission from [25]. Copyright 2020, PNAS.

Given the advantages of HTS, such as ultrahigh reaction temperatures of up to 3000 K, fast heating and cooling rates of 10^5^ K/s, and a peak temperature duration of nearly 60 ms, the thermodynamic limit of mixing immiscible elements can be surpassed [26,66]. Specifically, during this non-equilibrium HTS process, metal precursors, including thermodynamically immiscible metals, can be effectively decomposed into metal atoms, existing as a liquid alloy or plasma metal vapor under superhigh temperature conditions. During the cooling process, the ultrafast rates allow the metal mixtures to undergo rapid quenching, maintaining the high-temperature phase and anchoring the atomic arrangement with high entropy (Δ*S*), resulting in kinetically metastable phased HEAs (Fig. 5a). Due to the high-temperature fusion and fission, the intrinsic states such as disordered arrangement, defects and mixing of various elements can remain unchanged until ambient temperature is reached. Moreover, during the heating process, the metal atoms bind with carbon support firmly. This, combined with the inherent chemical stability of HEAs, leads to enhanced electrocatalytic activity and long-term stability. The HTS technique has the potential to produce metastable HEA nanoparticles, especially for the fine-tuning of elemental composition, creation of ultrafine-sized particles, and controlling of crystal phases, lattice distortions and strains due to the diversity of elemental radii. The particle aggregation, grain growth and phase separation are suppressed due to the rapid cooling rate of HTS during the preparation procedure [56]. To date, various HEA nanoparticles including PtPdRhRuCe, CoMoFeNiCu, PtCuNiCoFe and PtPdCoNiFeCuAuSn have been prepared as uniform solid solutions, which show superior activity and long-term stable and cost-effective catalytic performance (Fig. 5b) [26]. Specifically, Yao *et al.* employed a method involving the attachment of metal precursor salts to carbon fibers, resulting in the synthesis of HEA nanoparticles with compositions of up to eight elements. The process included HTS for 55 ms and heating/cooling rates of up to 10^5^ K/s. The practical viability of the resulting PtPdRhRuCe HEA nanoparticles was evaluated for ammonia oxidation catalysis, revealing an impressive conversion rate of nearly 100% and a selectivity exceeding 99%. Very recently, the high-temperature liquid shock (HTLS) method was applied to the synthesis of HEA alloys, such as PtCoNiRuIr nanoparticles, and delivered exceptional activity and long-term stability for HER, demonstrating low overpotentials of 18 mV and 408 mV at 10 mA cm^−2^ and 1 A cm^−2^, respectively, along with sustained stability over 10 000 CV cycles in a 0.5 M H_2_SO_4_ electrolyte [67]. Notably, the incorporation of a liquid medium in the HTLS method allowed for precise control over the morphology, size, dimension and crystal phase of the HEA nanoparticles through the introduction of reducing and capping agents. Furthermore, the application of Joule heating facilitated the maintenance of high-temperature reactions, enabling effective defect engineering in HEA nanoparticles, which can be challenging to achieve with conventional reported techniques.

Beside the synthesis of solid-solution HEAs, constructing heterojunctions is also an effective strategy to boost electrocatalysis. Shi *et al*. combined transition-metal-based HEA nanoparticles with noble metal clusters to manufacture efficient and stable heterostructures, referred to as HEA@Pt, via the galvanic displacement and HTS methods (Fig. 5d and e) [68]. Pt clusters exposed on the surface of the FeCoNiCu nanoalloy demonstrated excellent HER performance due to the modulation of surface-active sites, high dispersity, high entropy stabilization and core-shell synergistic interaction (Fig. 5f). Moreover, the high-throughput synthesis and screening of HEAs can be realized by combinatorial composition design and ultrafast Joule heating. Yao *et al*. proposed a high-throughput method involving the HTS process and the printing of precursor salts only [25]. The precursors with desired recipes are mixed in the liquid phase and deposited on CO_2_-activated carbon nanofibers (CA-CNFs, constructing surface defects on CNFs), and then HTS is used at 2000 K for ∼500 ms to realize rapid precursor decomposition and alloy formation (Fig. 5g). HEAs including PtPdRhRuIr and PtPdRhRuIrFeCoNi are manufactured, which demonstrate higher half-wave potentials and improved stability toward ORR, as compared to Pt/CNF (Fig. 5h). The HTS method shows great potential to build up a platform for efficient high-throughput synthesis for HEA catalysts. Very recently, Dong *et al*. applied the HTS method to synthesize free-standing high-entropy oxide (HEO) microparticles with a uniform elemental distribution, i.e. (Mn, Fe, Co, Ni, Cu, Zn)_3_O_4−_*_x_*, facilitating long-term electrochemical or thermochemical applications such as battery electrodes [69]. The key condition to obtain HEO microparticles is a longer heating time and loose-packed precursors, which can guarantee grain growth and prevent the formation of the interconnection of reduced domains [70]. Impressively, as reported by Cui *et al.*, high-entropy metal sulfide (HEMS, i.e. (CrMnFeCoNi)S*_x_*) nanoparticles can be synthesized via a pulse thermal decomposition method using Joule heating [71]. The as-prepared HEMS nanoparticles exhibited a synergistic effect among metal atoms that led to desired electronic states, enhancing the OER activity. Therefore, the HTS method can also be extended to the rapid synthesis of high-entropy materials in a broad compositional space, including but not limited to oxides, alloys, sulfides and phosphides.

### Metal compounds

Metastable metal compounds with unique electronic structures have gained increasing attention in the field of electrocatalytic reactions. Traditional synthesis methods for metal compounds, including solid-phase, sol-gel, co-precipitation and spray drying, involve prolonged heat treatment, multiple reaction steps, slow heating rates and sluggish reaction kinetics. In contrast, the HTS strategy offers a non-equilibrium approach with ultrafast heating and cooling rates, high reaction temperatures (up to 3000 K) and rapid reaction kinetics. Notably, HTS allows for a one-step synthesis of metal compounds within seconds [72,73]. Moreover, HTS is also an effective strategy for modulating the crystalline structure, grain size, surface and active electronic states of metal compounds. Specifically, by introducing oxygen vacancies (*V*_O_), the electronic structure of metal atoms can be optimized, resulting in improved electrical conductivity. *V*_O_ also plays a pivotal role in generating more active sites within the catalytic structure, fostering efficient charge transfer processes between the catalytic sites and reactant molecules. Consequently, this intricate manipulation substantially elevates the adsorption capacity of the electrocatalysts, enhancing electrocatalytic performance. For instance, air-assisted HTS is proposed to synthesize uniformly dispersed NiO@C/carbon cloth (NiO@C/CC) [74] and Fe_2_O_3_@C/CC [75] with abundant oxygen vacancies. The as-prepared metastable metal oxides, i.e. NiO@C/CC, showed excellent catalytic activity of 119.1 mA cm ^−2^ and robust durability in the electrooxidation of ethanol.

**Figure 6.**
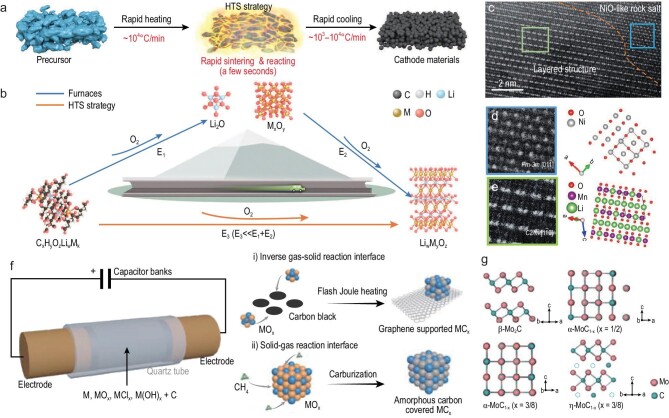
Rapid synthesis of metastable metal compounds by HTS technique. (a) Schematic illustration of the HTS manufacturing of cathode materials. (b) Reaction pathways of HTS and the conventional synthesis approach. (c) HAADF-STEM image of the Li-rich layered oxide/NiO heterostructure synthesized via the HTS method. (d) NiO phase and its atomic model. (e) Li-rich layered oxide phase and its atomic model. Reproduced with permission from [45]. Copyright 2022, Wiley-VCH GmbH. (f) Schematic illustration of the flashing Joule heating of carbides: (i) flashing Joule heating pathway; (ii) conventional carburization pathway. (g) Calculated crystal structures of carbides with different phases. The dashed circles represent carbon vacancies. Reproduced with permission from [76]. Copyright 2022, Nature Publishing Group.

Moreover, other metastable metal oxides employed as cathode materials for LIBs, such as LiMn_2_O_4_, LiCoO_2_, LiFePO_4_ and Li-rich layered oxide/NiO heterostructures, were synthesized through a promoted HTS method. Note that the heating rate and cooling rate were improved to 10^4^ K/min and 10^3^–10^4^ K/min, respectively (Fig. 6a and b) [45]. Meanwhile, the reaction process is controlled at high temperatures, facilitating rapid reaction kinetics. This non-equilibrium HTS process enables a one-step synthesis of the aforementioned metal oxides directly from the reacting precursors, including LiMn_2_O_4_, LiCoO_2_, LiFePO_4_ and Li-rich layered oxide/NiO heterostructures, within a few seconds. The as-synthesized metastable metal oxides showed abundant oxygen vacancies, dislocations, lattice strains and heterostructures (Fig. 6c–e). Importantly, these metal oxides showed excellent electrochemical performance when employed as cathode materials for LIBs. Moreover, HTS demonstrated a capacity to rapidly synthesize a variety of cathode materials with low energy consumption, allowing the high-throughput screening of novel cathode materials based on artificial intelligence. Using the synergistic electrical-thermal process of the flash Joule heating method, Tour *et al*. selectively synthesized metastable α-MoC_1-x_ and η-MoC_1-x_ by controlling the pulse voltage, demonstrating the phase engineering ability of the HTS technique (Fig. 6f and g) [76]. They also anticipate extending this highly energy-efficient method to the synthesis of other carbides and to the phase engineering of metastable carbides, providing a powerful method of carbide production [77,78].

### Carbon nanomaterials

**Figure 7.**
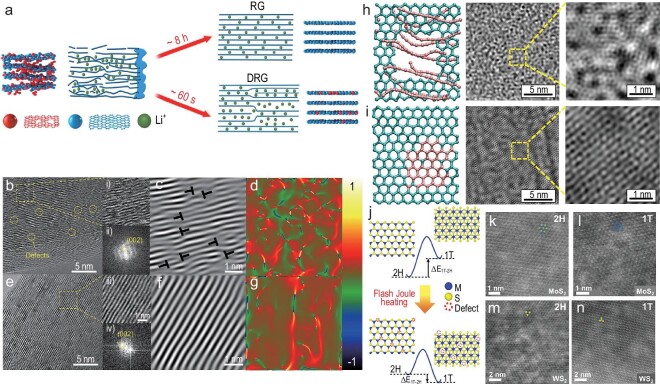
Carbon materials and other 2D materials. (a) Schematic illustration of the fabrication process of recycled graphite (RG) and defect-rich RG (DRG). (b and e) HRTEM images, enlarged images (see (i) and (iii)) and corresponding FFT patterns (see (ii) and (iv)) of DRG and commercial graphite, respectively. (c and f) IFFT images of (i) and (ii). (d and g) Strain distribution of e*_xy_*, corresponding to (c) and (f). Reproduced with permission from [80]. Copyright 2022, Springer. (h) Molecular dynamics simulated graphene sheets (top view) with hole defect, and the corresponding TEM images. (i) Molecular dynamics simulated graphene sheets (top view) after the hole defects were filled, and the corresponding TEM images. Reproduced with permission from [94]. Copyright 2019, Elsevier. (j) Schematic illustration of the energy level before and after the flash Joule heating process. (k and l) HAADF-STEM atomic images of flash MoS_2_ with 2H and 1T phases. (m and n) HAADF-STEM atomic images of flash WS_2_ with 2H and 1T phases. Reproduced with permission from [95]. Copyright 2021, American Chemical Society.

The reuse and recycling of spent graphite anode materials for LIBs is crucial to mitigating the greenhouse effect caused by excessive emissions of CO_2_, and to reducing energy wastage with a view to full decarbonization and a carbon-neutral society. Traditionally, recycled graphite undergoes extended heating within a tube furnace for several hours, but this approach generates only a few defects, limiting the improvement of its capability [79]. However, the spent graphite can be transformed into defect-rich recycled graphite (DRG) in just 60 s using the non-equilibrium HTS strategy under an Ar atmosphere at 1773 K (Fig. 7a) [80]. Owing to the ultrafast heating and cooling process, the graphite can preserve both layered structures and abundant defects from the charging and discharging cycles (Fig. 7b–g, and i–iv). In addition to retaining the original vacancy defects of spent graphite, HTS-prepared DRG incorporates a large quantity of dislocations, which greatly facilitates the thermodynamic phase transformation and kinetic diffusion of Li ions. Impressively, the as-obtained DRG anode material delivered a remarkable charging specific capacity of 323 mAh/g at a high current density of 2 C. Defect engineering offers promising perspectives, and orchestrates the atomic-scale re-graphitization of spent graphite, rendering it viable for integration into batteries characterized by exceptional performance and elevated throughput [80,81]. This HTS method is important in the landscape of battery recycling, not only due to its capacity to address ecological concerns associated with spent anode materials but also for its role in propelling advancements towards sustainable energy-storage solutions.

### TEM grids for metastable materials

Some categories of metastable or amorphous materials are sensitive to electron beam irradiation or exposure to air, such as electrodes in LIBs, chiral materials with ligands, or amorphous metal compounds [82–85]. To fully characterize the aforementioned materials, low-dose transmission electron microscopy (TEM) and cryogenic electron microscopy (cryo-EM) are usually applied to expose materials to less irradiation to avoid the knocking out of atoms from the surface [86,87], preventing phase transformation or damage to the target materials during characterization [88,89]. Moreover, to compensate for the sacrifice of signal-to-noise ratio (SNR) resulting from the low electron dose applied, TEM grids with excellent electric/thermal conductivity, mechanical strength and minimal background noise are desirable for the detailed characterization of metastable or amorphous materials.

Remarkably, 2D nanomaterials, such as graphene, graphene oxide (GO), rGO, graphdiyne, hexagonal boron nitride (h-BN), transition metal dichalcogenide (e.g. MoS_2_), MXene and metal nanosheets, are promising candidates for supporting films for TEM grids due to their excellent physicochemical properties [90–93]. Worth noting is the successful application of the HTS technique in fabricating highly dense and defect-free 2D graphene film with excellent electrical conductivity (2209 S/cm) and thermal conductivity (863 W/mK), by rapid thermal heating at ultrahigh temperatures of up to 2700 K [94]. Different from conventional thermal treatment in a graphite furnace, the HTS strategy applies Joule heating to induce a superhigh temperature within defect-prone zones of the graphene nanosheets, where the electrical resistance is higher. The adjacent graphene nanosheets or the defect-rich areas can form crosslinks by self-healing thermal reduction, so as to construct highly dense and defect-free graphene films (Fig. 7h and i). Furthermore, HTS is also used to realize the phase transformation and defect construction of other 2D materials such as MoS_2_ membranes, which can tune the binding force between 2D MoS_2_ supporting films and the target nanoparticles or proteins. Tour *et al.* successfully converted MoS_2_ and WS_2_ from 2H phases to 1T phases within milliseconds via flash Joule heating (Fig. 7j–n) [95]. The phase conversion ratio can also be determined by modifying the reaction duration and additives. During the conversion process, defects like vacancies can be generated as well. The phase diversity and defects formation may result in a change in electrostatic adsorption between the 2D film and oxygenated functional groups, which may affect the binding force between the film and nanoparticles or proteins. Potentially, the HTS technique could be applied to the preparation of different functionalized supporting films for a TEM grid, which might be important in the structural engineering of 2D materials for TEM characterization, and even benefit cryo-EM methodology and structural biology.

## CONCLUSION AND PERSPECTIVES

In this report, the non-equilibrium HTS technique is systematically described with typical characteristics, i.e. ultrafast heating or cooling rates, short dwelling time and superhigh temperature. Furthermore, the thermodynamic and kinetic mechanisms of the HTS synthesis system for metastable materials are explained. In addition, recent advances in versatile metastable nanomaterial manufacturing via the high-throughput HTS strategy in various electrocatalytic and energy-storage and conversion applications are systematically presented, including uniformly dispersed mono-/bimetallic nanomaterials, HEAs, metal compounds, carbon nanomaterials and 2D supporting films for TEM grids. Note that the synthesis of metastable materials via high-throughput HTS is an attractive research direction, with its essence lying in comprehensive high-throughput characterization. For electrocatalysis, despite the utilization of microscanning electrochemical workstations, there exists a need for more efficient and advanced characterization methods to enhance the understanding and evaluation of metastable electrocatalysts. Promising avenues to be explored in the near future are as follows:

- The high-throughput and large-scale industrial production of metastable nanomaterials has great potential to efficiently screen suitable elemental components, crystal structures and defects to produce excellent catalysts for various applications. Combined with artificial intelligence to screen the reaction system, theoretic calculations to explain the kinetic and thermodynamic mechanisms, high-throughput electrocatalytic tests to select catalysts with high performance, machine learning, and big data, we believe this innovative HTS approach holds the key to unlocking a wide array of smart applications, marking a transformative step forward in the realm of materials science.
- The HTLS method directly employs Joule heating in the liquid-state reaction system consisting of reacting precursors, capping agents and reducing agents in solution. It is straightforward to modulate the size, dimension, facet, morphology and crystal phase of nanostructures due to a variety of tunable parameters including capping agents, solvents and dispersants in the reaction system. On the one hand, the HTLS method can heat the reaction system to high temperatures of up to several hundred Kelvins within several seconds, and maintain this for dozens of minutes. This overcomes the limitations of the traditional liquid-phase method, such as slow heating or cooling rates, low peak temperature and time-consuming processes. On the other hand, due to the introduction of a liquid-phase environment, the HTLS method can provide more uniform reaction processes, achieving simple and precise control over the morphology and phase of final products, as compared to the HTS approach. Therefore, the HTLS method shows remarkable advantages such as versatile reaction systems, high reaction temperature and high efficiency, which are beneficial for the kinetic modulation and high-speed construction of nanomaterials with refined size, distinct atomic arrangement and high-density defects. The catalysts synthesized via the HTLS method exhibit abundant uncoordinated sites and dangling bonds, enhancing their electrocatalytic performance.
- Multicomponent nanocatalysts, e.g. HEAs, are promising for the selective conversion of CO_2_ to multicarbon products. Nevertheless, several challenges persist in the HTS synthesis of HEA nanoparticles for catalytic applications, including precise control over the compositional homogeneity of HEAs, a comprehensive understanding of the impact of kinetics control over the HEA structures, and the optimization of catalytic activity and stability of HEA catalysts. Furthermore, the kinetic and thermodynamic mechanisms of HEA structural evolution during the HTS process are still to be investigated. Systematically addressing these challenges is essential to advancing the rational design and practical utilization of HEA nanoparticles in catalytic applications. Cu-based HEA nanostructures containing five or more active metals can be synthesized using the ultrafast HTS strategy. These multicomponent catalysts exhibit metastable or heterophase structures, where Cu atoms are stabilized by other metals. By tuning the crystal phase, defects and components of Cu-based HEAs, we can explore the effect of the crystal phase, surface/interface and elements on CO_2_ conversion with high-value carbon products. Note that rare metal is an alternative candidate for forming HEA nanostructures as well. By modulation of the *d*-band center and hybridized orbits (*d* or *f*) of the selected elements, the electronic structures and synergistic effects among various metal compounds in HEA can be rationally designed, enhancing the performance for tandem catalysts in a variety of electrocatalysis situations. For instance, a Cu- and Ce-based HEA library is ideal for tandem CO_2_ reduction to convert CO_2_ into higher hydrocarbon fuels.
- Combined with *in-situ* observation strategies, we can deeply understand structure evolution and the chemical state of real active sites and reaction intermediates. Specifically, *in-situ* characterizations, such as *in-situ* TEM, X-ray diffraction (XRD), X-ray photoelectron spectroscopy (XPS) and Raman spectroscopy, play a crucial role in monitoring the precise evolution of metastable phases and intermediates during the fabrication process. This capability facilitates the optimization of synthesis conditions, including temperature, pressure and precursor composition, to tailor the properties of metastable materials. Furthermore, *in-situ* techniques contribute to identifying reaction pathways, enhancing the design of more efficient and controllable synthesis routes. In electrocatalytic applications, employing *in-situ* characterizations such as *in-situ* X-ray absorption spectroscopy (XAS) and spectroscopy enables the direct observation and evaluation of structural and property changes in catalysts during electrochemical processes. Real-time monitoring of metastable materials under working conditions, while mimicking realistic environments, proves crucial for designing effective electrocatalysts. This methodology provides valuable insights into structural changes, surface reactivity and active site evolution during electrocatalysis, thereby benefiting the structural engineering of metastable materials. This holistic approach contributes to a more comprehensive understanding of the structure–function relationship, facilitating the rational design of metastable materials for improved electrocatalytic performance. In summary, *in-situ* characterization techniques serve as powerful tools in both the synthesis and electrocatalytic application of metastable materials, providing real-time insights into dynamic processes and enabling precise control over synthesis conditions.
- Large-scale 2D materials such as graphene fabricated by the CVD method, GO, rGO, MoS_2_ and MXene are easily oxidized or adsorbed by impurities, or contain defects. The HTS technique has been proven to heal the defects of graphene by high-energy Joule heating. Moreover, the HTS technique is promising with regard to removing impurities on the surface of 2D materials, thereby improving their crystallinity and mechanical strength. Importantly, such 2D materials have the potential to be used as supporting films for TEM/cryo-EM grids or liquid cells, thereby boosting the development of material science and structural biology.
